# Supplementary material for: An epigenetic clock to estimate the age of living beluga whales
Source: Evol Appl. 2021 Feb 3;14(5):1263–73. doi: 10.1111/eva.13195 (PMC8127720; doi:10.1111/eva.13195)

**Figure S1.** Photos taken in the field of (A) whale MML-RA170907-B01aged by the beluga epigenetic clock at approximately 11 years old and (B) whale MML-RA170904-B01 aged by the beluga epigenetic clock at approximately 27 years old. The top photograph shows a younger, grey whale characteristic of juveniles while the bottom photograph shows a mature adult whale with a bright white coloration. Photo credits: Paul Wade. NMFS MMPA/ESA Permit # 20465.

(A)


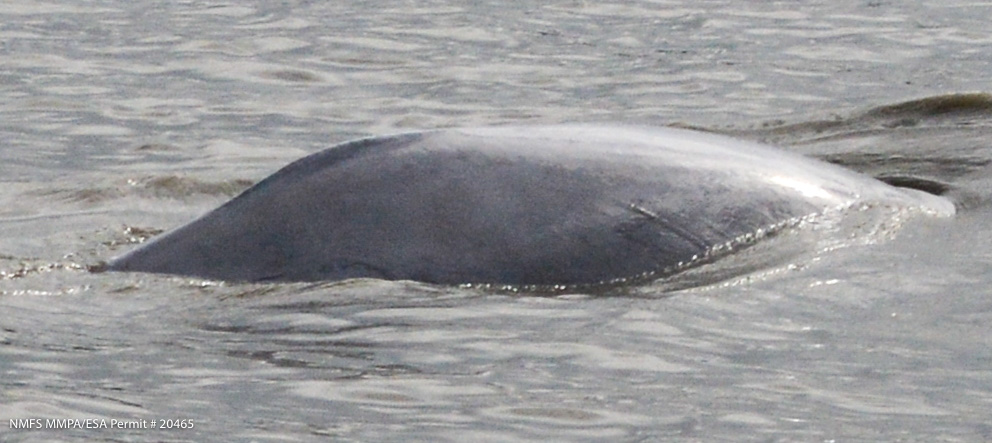


(B)


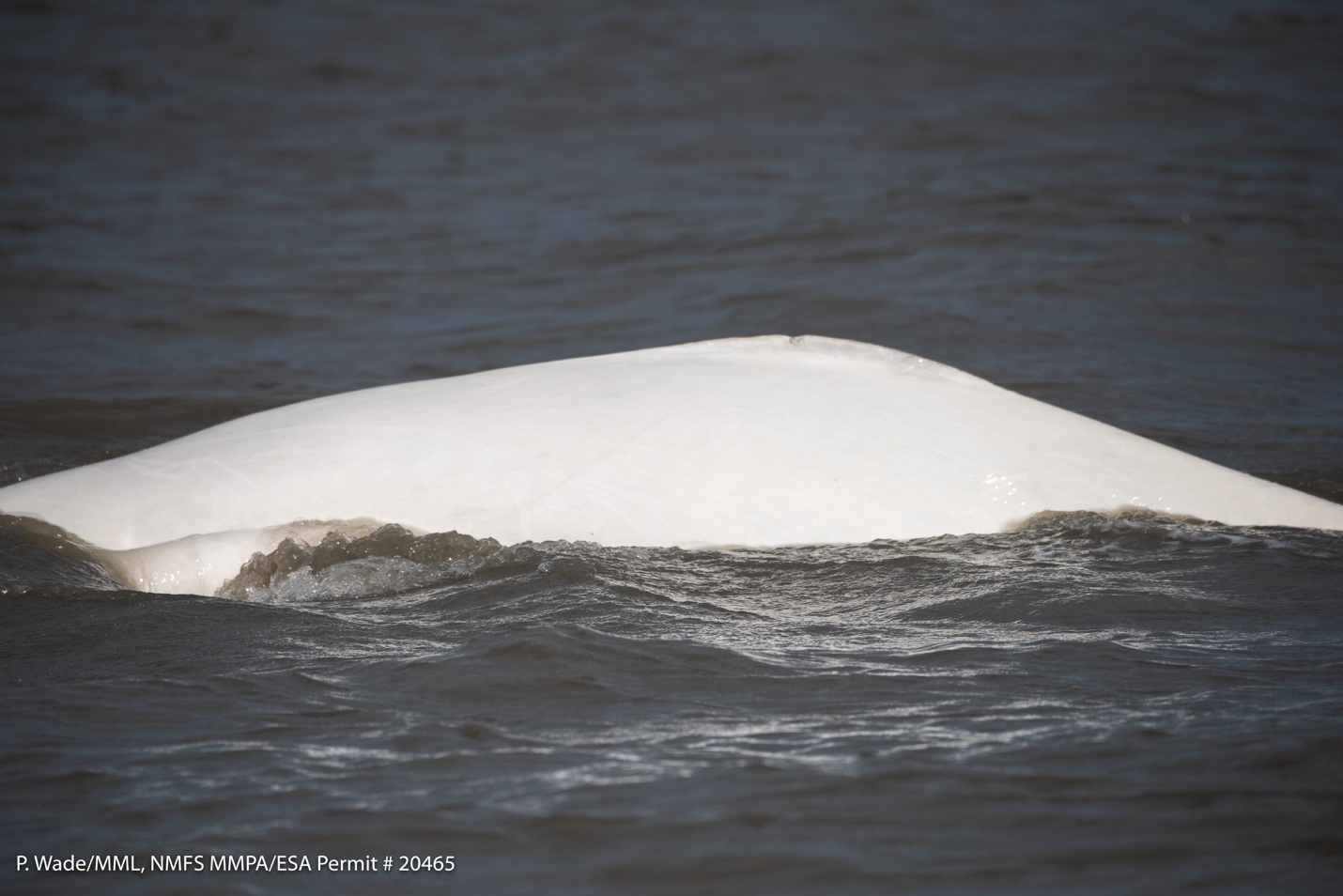

Supplement: Supplementary file 1 — Fig S1 [file EVA-14-1263-s003.docx]
